# Primary Exercise Headache

**DOI:** 10.1007/s11910-020-01028-4

**Published:** 2020-04-15

**Authors:** Parth Upadhyaya, Arathi Nandyala, Jessica Ailani

**Affiliations:** Department of Neurology, Medstar Georgetown University Hospital, 3800 Reservoir Road NW, 7PHC, Washington, DC 20007 USA

**Keywords:** Primary exercise headache, Benign exertional headache, Primary exertional headache, Intracranial hypertension, Indomethacin

## Abstract

**Purpose of Review:**

Primary exercise headache has gone through many descriptors in the past but generally is a headache that is precipitated by strenuous exercise without significant intracranial pathology. Its presentation can remain vague, often confused with other primary and secondary headache disorders and thus undertreated. This review aims to discuss primary exercise headache in the context of epidemiology, presentation, pathophysiology, differential diagnosis, and treatment.

**Recent Findings:**

Two large epidemiological studies in Iran and Japan have further characterized a predilection for female patients, comorbidity with migraine, and frequent bilateral nature of headache.

**Summary:**

While large-scale epidemiological studies have aided in further characterization and determining varying prevalence, a lack of randomized clinical trials in the treatment of primary exercise headache remains. Indomethacin and beta-blocker use remain the mainstays of treatment based on case series with several case reports that urge caution when diagnosing said headache.

## Introduction

Primary exercise headache (PEH) is a type of headache with no known intracranial pathology but occurs during or immediately after exercise. One of the first studies examining this type of headache was in 1968 [1]. Prior to this, it had been thought that an intracranial lesion had to be present, often leading to a lengthy workup [1]. Since then, other names that have been used include primary exertional headache and benign exertional headache.

The prevalence of PEH varies greatly from one study to another; however, among all headaches, it is relatively rare. In one study of 975 subjects from the Danish National Central Person Registry, 1% of the general population was found to have benign exertional headache according to the IHS diagnostic criteria [2]. In another prospective study of over 6000 patients seen over 10 years for headaches, only 1.5% were considered provoked and 11% of those were considered exertional [3]. Patients were generally younger, with an average age of onset 40 and predominantly male [3]. A large epidemiological study looking at over 1800 patients found the prevalence of EH to be 12.3%, with a slight female predominance [4]. Another study focused solely on cyclists competing in a strenuous bike race found a PEH prevalence of 26% with a declining prevalence with increasing age [5]. The higher prevalence of PEH may be secondary to the population of the study, dehydration, or body temperature. In a 2015 epidemiology study of 2076 patients in Iran, the 1-year prevalence of PEH was seen at 7.3%, with a significant preponderance for females (10% vs 5.4% men) and a mean age of 32 years (± 12 years) [6•]. A 2015 Japanese study evaluated 2546 patients with headache and identified 30 patients with PEH using the current guideline classifications [7•]. They also identified that PEH was more common in women (1.77% vs. 1.19%, 0.82% in men) and found a mean age of 43 years of onset [7•]. Headaches were located bilateral in 23 of 30 patients, occipital (16 patients), or frontal (10 patients) and lasted from 5 min to 12 h [7•]. PEH was comorbid with migraine without aura in most patients (20 out of 30), headache with sexual activity (7 patients), or cough headaches (5 patients) [7•].

## Symptomatology/Presentation and Diagnosis

Clinical descriptions of patients with PEH in the literature is minimal and largely from small case series. From these descriptions, patients present with a bilateral pulsatile or throbbing head pain without nausea or vomiting. These episodes can last anywhere from 5 min to 48 h and always precipitated by strenuous exercise [1, 2, 7, 8]. The International Classification of headache disorders 3rd edition defines PEH as patients having at least two headache episodes that last less than 48 h and precipitated by or during strenuous exercise and must also not be better accounted for by another ICHD-3 diagnosis [9].

## Differential Diagnosis

While PEH is often a benign diagnosis, secondary etiologies like reversible cerebral vasoconstriction syndrome (RCVS), cervical artery dissection, idiopathic intracranial hypertension, cardiac cephalgia, and pheochromocytoma may present with a similar phenotype. Paulson published a case report of a young female swimmer with reported exertional headaches, who collapsed during practice, found to have pulmonary edema and ultimately diagnosed with pheochromocytoma [10]. Wei published a case series describing cardiac cephalgia, emphasizing the importance of ruling out cardiac disease in patients with exertional headaches and risk factors [11]. For example, Curter et al. described a patient with new-onset headache, without associated chest pain or EKG changes, who underwent myocardial perfusion imaging (MPI) as part of a cardiac evaluation due to exertional nature of pain. This patient underwent cardiac stenting after discovering cardiac ischemia; post-procedure, the patient no longer experienced an exertional type of headache [12]. Based on these cases, one should consider critical points of history including coronary artery disease, recent head or neck trauma, and malignancy as etiologies to first exclude. Exertional headache with associated fever, shortness of breath, chest pain, or focal neurologic deficits should point to cardiac, pulmonary, neurologic, or systemic involvement. Evaluation should include MRI imaging of the brain and cervical spine along with dedicated vessel imaging of the head and neck as per history. The presence of cardiac risk factors may warrant further evaluation with EKG and exercise stress tests to evaluate for cardiac ischemia. See Table 1 for differential evaluation of exertional type headache.

**Table 1:** Differential evaluation of exertional type headache

Differential diagnosis	Symptoms	Tests/imaging to consider
Reversible cerebral vasoconstriction syndrome	Peaks in intensity within seconds Hypertension	MRI cerebral angiography
Cervical artery dissection	Horner syndrome Bruit Tinnitus Neuropathy Numbness/weakness	MRI MRA head and neck
Idiopathic intracranial hypertension	Papilledema Tinnitus Diplopia Temporal visual field loss Abducens palsy	MRI LP Ophthalmologic evaluation
Cardiac cephalgia	Chest pain Medical history significant for coronary artery disease, HTN, HLD Headache resolves with medical treatment or revascularization	EKG Troponin Exercise stress test
Pheochromocytoma	Sweating Tachycardia Paroxysmal elevations in blood pressure Cardiomyopathy	Free plasma metanephrine level

## Pathophysiology and Etiology

Dysregulated cerebrovasculature has been theorized to play a role in the pathophysiology of PEH [13]. Tinel describes how incompetent internal jugular valves in the context of Valsalva maneuvers can increase intrathoracic pressure and decrease cerebral venous drainage, thus playing a role in the cause of PEH [13]. Retrograde flow from incompetent venous vasculature may lead to a transient increase in blood flow, causing an increase in intracranial pressure leading to head pain [14]. One study aimed to assess retrograde venous flow in 20 exertional headache patients and 40 controls during Valsalva maneuvers [14]. Seventy percent of exertional headache patients demonstrated venous retrograde flow and thus valvular incompetence [14]. Dysfunction of normal autoregulation of cerebrovasculature has been discussed in exertional headache and in several other primary headache disorders [14]. The ability to maintain perfusion of tissue without significant fluctuation in the face of shifting pressures and metabolic demands could play a role in exertional headache [15]. Physical exertion itself under normal physiologic circumstances leads to rapid changes in blood pressure in order to maintain perfusion, with changes to a resting state leading to its reversal [15]. Transient spikes in blood pressure via exertion in a system without adequate autoregulation could lead to similar increases in intracranial pressure and thus pain [15].

## Management

There are a lack of randomized clinical trials for PEH to suggest protocoled management for the disorder; guidance for treatment is based on case series, case reports, and expert opinion statements. In one early study of 15 patients with PEH, successful control of headache with continuous use of indomethacin was noted [16]. The literature describes a therapeutic dose ranging from 25 to 150 mg taken before planned strenuous activity [16]. The ability of indomethacin to reduce cerebral blood flow and thus reduce intracranial pressure, as suggested by one review on the treatment of increased ICP in traumatic brain injury, lends itself to previous theorized pathophysiologic mechanisms of pain in PEH [17]. Overlap between other headache disorders can lend alternate therapeutic considerations as in the case of headache with sexual activity. In one case series of 45 patients, 9 patients experienced both PEH and headache with sexual activity within 72 h of each other. Eighteen patients in total were seen to have a comorbidity of both headache types. In terms of management, this study found benefit in a cohort of patients with sexual headache with either propranolol at 40 to 80 mg daily or atenolol 50 mg. Given the significant overlap of both headache types, a possible final common pathophysiological pathway may be present and lend benefit of beta-blockade to primary exercise headache [18].

## Cases

### Here we Present Two Cases of PEH to Review Diagnosis and Management

#### Case 1

A 44-year-old male presents to a neurology outpatient clinic for evaluation of headache during his daily commute home. He explains that the headache occurs shortly after walking up a steep hill. The headache rapidly progresses from the left occipital region of his head to bilateral occiput. The headache resolves upon waking the following morning but consistently returns, after completing the same commute, day after day. The patient remarks this has occurred once before during sexual relations but not since. He denies any nausea, blurred vision, or prior history of headaches. His past medical history is negative except for a recent motor vehicle accident. His family history is significant coronary heart disease, stroke, and diabetes.

In this patient, given his history of MVA and significant family history of heart disease, an MRA of his head and neck was obtained and primary evaluation of his cardiac function was done. His imaging revealed no dissection or stroke. His cardiac evaluation found hyperlipidemia now treated with a statin, EKG without significant signs of ischemia, and normal exercise stress test. The decision made with his primary physician to start low-dose propranolol helped reduce the length and severity of his headaches.

#### Discussion Case 1

This patient presents with headache after prolonged strenuous exercise and once even with sexual activity. This case illustrates the overlap in patients with exertional component to pain to evaluate for appropriate alternative etiologies. In this instance, evaluating his cardiac risk factors for angina and recent trauma leads to dissection. Treatment to manage cardiac risk factors and headache makes propranolol an appropriate choice. If headaches were not improved, up-titration of propranolol or the use of indomethacin as needed prior to strenuous activity could be reasonable treatment strategies.

#### Case 2

A 26-year-old female presents to her primary care’s office with a chief complaint of headache. The pain started 1 month prior while performing heavy weightlifting in preparation for a competition. She states that the headache resolved the following day with NSAID use. She reports that now after every weightlifting session, she suffers a crushing bilateral headache lasting 2 h. Her primary physician started her on low-dose atenolol daily and indomethacin prior to weightlifting. This provided minimal relief after a 2-month trial. The patient subsequently presented to the emergency department for headache and urine toxicology was positive for cocaine. Cerebral angiogram completed found a string of bead appearance and diagnosis of reversible cerebral vasoconstriction syndrome (RCVS). The patient was counseled on cocaine abuse and started on calcium channel blocker with modest improvement in headache.

#### Discussion Case 2

This case demonstrates the importance of the evaluation of secondary etiologies of headache and re-evaluation in the treatment of refractory cases of suspected primary exercise headaches. While the headache was precipitated by exercise, the use of illicit stimulants precipitated RCVS which may mimic primary exercise headaches.

## Conclusion

Primary exercise headache presents during or after strenuous activity with pain lasting upwards of 48 h. Investigations of secondary pathologies that may also worsen with exertion or manifest in a temporal manner, including cerebrovascular and cardiac etiologies, should be evaluated before diagnosis. Patients that fail to respond with beta-blockade or indomethacin should warrant reassessment for secondary causes.
